# Variations of the *UNC13D* Gene in Patients with Autoimmune Lymphoproliferative Syndrome

**DOI:** 10.1371/journal.pone.0068045

**Published:** 2013-07-01

**Authors:** Maurizio Aricò, Elena Boggio, Valentina Cetica, Matteo Melensi, Elisabetta Orilieri, Nausicaa Clemente, Giuseppe Cappellano, Sara Buttini, Maria Felicia Soluri, Cristoforo Comi, Carlo Dufour, Daniela Pende, Irma Dianzani, Steven R. Ellis, Sara Pagliano, Stefania Marcenaro, Ugo Ramenghi, Annalisa Chiocchetti, Umberto Dianzani

**Affiliations:** 1 Department of Pediatric Hematology Oncology, Meyer Children Hospital, Firenze, Italy; 2 Interdisciplinary Research Center of Autoimmune Diseases (IRCAD), “A. Avogadro” University of Eastern Piedmont, Novara, Italy; 3 Department of Health Sciences, “A. Avogadro” University of Eastern Piedmont, Novara, Italy; 4 Department of Translational Medicine, "A. Avogadro" University of Eastern Piedmont, Novara, Italy; 5 Istituto Giannina Gaslini, Genova, Italy; 6 IRCCS AOU San Martino-IST, Genova, Italy; 7 Department of Biochemistry and Molecular Biology, University of Louisville, Louisville, Kentucky, United States of America; 8 Department of Pediatrics, University of Torino, Torino, Italy; University of Birmingham, United Kingdom

## Abstract

Autoimmune lymphoproliferative syndrome (ALPS) is caused by genetic defects decreasing Fas function and is characterized by lymphadenopathy/splenomegaly and expansion of CD4/CD8 double-negative T cells. This latter expansion is absent in the ALPS variant named Dianzani Autoimmune/lymphoproliferative Disease (DALD). In addition to the causative mutations, the genetic background influences ALPS and DALD development. We previously suggested a disease-modifying role for the perforin gene involved in familial hemophagocytic lymphohistiocytosis (FHL). The *UNC13D* gene codes for Munc13-4, which is involved in perforin secretion and FHL development, and thus, another candidate for a disease-modifying role in ALPS and DALD. In this work, we sequenced *UNC13D* in 21 ALPS and 20 DALD patients and compared these results with sequences obtained from 61 healthy subjects and 38 multiple sclerosis (MS) patients. We detected four rare missense variations in three heterozygous ALPS patients carrying p.Cys112Ser, p.Val781Ile, and a haplotype comprising both p.Ile848Leu and p.Ala995Pro. Transfection of the mutant cDNAs into HMC-1 cells showed that they decreased granule exocytosis, compared to the wild-type construct. An additional rare missense variation, p.Pro271Ser, was detected in a healthy subject, but this variation did not decrease Munc13-4 function. These data suggest that rare loss-of-function variations of *UND13D* are risk factors for ALPS development.

## Introduction

The lytic granules of cytotoxic T cells (CTL) and natural killer (NK) cells contain perforin and granzymes, which are released on the target cell surface and induce its death[1]. . The exocytosis mechanism of the lytic granules is not fully understood, but it involves machinery composed of several proteins including Munc13-4, Munc18-2, and syntaxin11 [2]. Deficiencies of perforin function are responsible for familial hemophagocytic lymphohistiocytosis (FHL), an autosomal recessive disease characterized by bouts of prolonged fever, hepatosplenomegaly, and cytopenia due to defective function of CTL and NK cells. FHL has been ascribed to defective clearance of virus-infected cells leading to cytokine and effector cell overproduction with massive tissue damage [3]. Approximately 40% of FHL cases (FHL2) are due to mutations of the perforin gene (*PRF1*), with another 40% (FHL3) due to mutations of the Munc13-4 gene (*UNC13D*). Moreover, a small number of patients with FHL have been found to harbor mutations of *STX11*, encoding Syntaxin-11 (FHL4), or *STXBP2*, encoding Munc18-2 (FHL5) [4].

Autoimmune lymphoproliferative syndrome (ALPS) is another genetic lymphoproliferative disease and is characterized by lymphadenomegaly and/or splenomegaly, due to polyclonal accumulation of lymphocytes, and peripheral expansion of CD4/CD8 double-negative (DN) T cells [5–10]. In addition, patients often display autoimmune manifestations that predominantly involve blood cells and are predisposed to lymphomas in adulthood [11]. ALPS is due to defective function of the Fas/Apo-1 (CD95) death receptor, inducing apoptosis of the Fas-expressing cell upon binding with Fas ligand (FasL) [12,13]. Activated lymphocytes express Fas and the Fas/FasL interaction is involved in shutting off immune responses, lymphocyte lifespan regulation, and maintenance of peripheral tolerance [14,15]. Moreover, the Fas pathway is an additional weapon, reinforcing the perforin system in the cytotoxicity mediated by CTL and NK cells because these cells express FasL and induce apoptosis of target cells expressing Fas. In most patients (ALPS-FAS), ALPS is due to mutations of the Fas gene (*FAS*), but a small number of patients (ALPS-FASL and ALPS-CASP10) carry mutations in the genes encoding FasL (*FASL*) or caspase-10 (*CASP10*), a downstream effector in the Fas/FasL pathway. As a substantial proportion of ALPS patients (ALPS-U) lack mutations in *FAS*, *FASL*, and *CASP10*; it seems likely that mutations in unknown genes encoding other downstream components of the Fas cell death pathway may give rise to the additional ALPS cases [9,10]. We have also described an incomplete form of ALPS showing defective Fas function, autoimmunity, and lymphoproliferation, but lacking the expansion of DN T cells. This variant form has been named Dianzani Autoimmune Lymphoproliferative Disease (DALD) by Victor McKusick (OMIM # 605233) [10,16–18]. Patients with DALD did not display mutations in *FAS*, *FASL*, or *CASP10*, but most of the parents displayed a defect in the Fas pathway. These data suggest that mutations in genes encoding downstream effectors of the Fas pathway may also give rise to DALD.

In addition to the Fas defect, the clinical presentation of ALPS also appears to be influenced by modifier genes. In mice, a disease displaying features of ALPS has been reported for MRL *lpr/lpr* and *gld/gld* mice, carrying mutations of *FAS* and *FASL*, respectively. Disease presentation in these mice is dramatically affected by strain background, with strains other than MRL showing much milder phenotypes when homozygous for either *lpr* or *gld* mutations [12,19]. Similar background effects likely explain the incomplete penetrance of ALPS mutations in humans [20]. Most ALPS patients are heterozygous for the *FAS* mutation, but parents carrying the same mutation are generally healthy. The same observation is true in DALD, where parents typically display defective Fas function, but are otherwise healthy [17,18]. This observation indicates that mutations in genes of the Fas pathway may be necessary but not sufficient for ALPS development and variations in one or more additional genes may influence disease presentation [9].

In previous works, we correlated certain variants of the perforin gene (*PRF1*) with ALPS/DALD development and suggested that mild heterozygous variations of *PRF1* incapable of inducing FHL may act as susceptibility genes for ALPS/DALD development in subjects displaying defective Fas function [21,22]. The aim of this work was to extend this observation to the *UNC13D* gene, looking for variations in ALPS and DALD patients and assessing its potential role as a disease-modifier gene. We found that loss-of-function variations of *UNC13D* are relatively frequent in patients with ALPS, suggesting that it may influence the presentation of this lymphoproliferative disorder.

## Materials and Methods

### Patients

We analyzed 41 unrelated Italian patients, 21 with ALPS and 20 with DALD. All patients were diagnosed at the Pediatric Department of the University of Turin using criteria established at the 2009 ALPS NIH International Workshop [10]. *FAS* (NCBI ID: 355) and *CASP10* (ID: 843) were sequenced in all patients as reported previously [16,17]. Among the ALPS patients, 7 carried heterozygous mutations of *FAS* (ALPS-FAS), 14 did not carry any known mutation (ALPS-U). None of the patients fulfilled the diagnostic criteria for FHL. A total of 61 healthy individuals were used as controls for *UND13D* sequencing, and a second cohort of 100 healthy controls were used to genotype the rare variations. Moreover, *UNC13D* was sequenced in 38 patients with Multiple Sclerosis (MS) from the MS Center of the "Amedeo Avogadro" University of Eastern Piedmont (Novara). The study was planned according to the guidelines of the local ethical committee, Azienda Ospedaliera della Carità, of Novara that approved the study (Protocol 106/CE). For the patients followed at Paediatric Department of the University of Torino, a written informed consents was signed by the patients, or by the parents if they were minors.

### Fas function assay

Fas-induced cell death was evaluated on T cells obtained by activating peripheral blood mononuclear cells (PBMC) with phytohemagglutinin (Sigma, St Louis, MO, Canada) at days 0 (1 µg/mL) and 12 (0.1 µg/mL) and cultured in RPMI 1640 plus 10% fetal calf serum and recombinant IL-2 (rIL-2, 2 U/mL) (Sigma). Fas function was assessed 6 days after the second stimulation (day 21). Cells were incubated with control medium or anti-Fas monoclonal antibody (mAb) (CH 11, 1 µg/mL) (Millipore, Billerica, MA) in the presence of rIL-2 (1 U/mL) to minimize spontaneous cell death. Cell survival was evaluated after 18 hours by counting live cells by the trypan blue exclusion test. Assays were performed in duplicate. Cells from 2 healthy donors were included in each experiment as positive controls. The results were expressed as percent specific cell-survival, calculated as follows: (total live-cell count in the assay well/total live-cell count in the control well) X100%. Fas function was defined as defective when cell survival was less than 82% (the 95^th^ percentile of data obtained from 200 healthy controls) [17,18].

### UNC13D sequencing

Genomic DNA was isolated from peripheral blood samples using a BioRobot® EZ1 Workstation (Qiagen, Jesi, Italy). Exons and intron-exon boundaries of *UNC13D* (ID: 201294), were amplified and directly sequenced in both directions with the BigDye® Terminator Cycle Sequencing Ready Reaction Kit (Applied Biosystems, Foster City, CA, USA). Primers are available upon request. Sequences were analyzed and compared with the reported gene structure. The missense variations identified in patients were also assessed in parents.

Allele expression was evaluated in total RNA extracted from PBMC of heterozygous donors. RNA was reverse transcribed into cDNA with the ThermoScript^TM^ RT PCR System (Invitrogen, Burlington, ON, Canada) and the exons containing the *UND13D* variations were amplified. PCR products were subcloned into the pGEM-T vector (Promega Corporation, Madison, WI, USA) and transformed into TOP10 *E. coli* competent cells (Invitrogen). In each selected patient, we screened 30 independent colonies; plasmid DNA was extracted with a QIAPrep Spin miniprep Kit (QIAGEN GmbH, Hilden, Germany) and sequenced.

### Functional analysis of the variations

Munc13-4 cDNA (ImaGenes, BioDiscovery, Inc. Suite CA, USA) was subcloned into the pcDNA 3.1 expression vector (Invitrogen), and the Sv5 tag was added at the 5′ end by PCR. The mutants were created in the Munc13-4 wild-type construct by PCR and then transfected into the HMC-1 human mast cell line, originally established from the peripheral blood of a patient with mast cell leukemia [23] and kindly provided by C. Dianzani. Cells were transfected by Amaxa Cell Line Nucleofactor Kit V (Lonza, Basel, Switzerland), according to the manufacturer’s instructions. Briefly, 4 µg of each construct were co-transfected with 1 µg of the pEGFP vector (Invitrogen). Transfection efficiency was analyzed by cytofluorimetric evaluation, and determined by calculating the % of GFP expressing cells. To investigate Munc13-4 expression levels, cells were lysed and proteins resolved by gel electrophoresis were analyzed using mAb to Sv5 and actin (Santa Cruz Biotechnology, Inc. Santa Cruz, CA, USA). Immunoreactive proteins were visualized with HRP-conjugated goat anti-mouse IgG (Sigma).

To investigate primary granule exocytosis, transfected HMC-1 cells (1x10^6^/ml) were incubated in Tyrod Buffer (Hepes 10 mM pH=7.4, NaCl 173 mM, KCl 2.9 mM, NaHCO_3_ 12 mM with 1.6 mM CaCl_2_ and 5 mM Glucose) and stimulated with 10^-6^ M formyl-methionyl-leucyl-phenylalanine peptide (fMLP, Sigma) at 37°C for 10 min. Exocytosis was then assessed on the GFP-expressing cells by cytofluorimetric analysis of CD63 expression, using the mean fluorescence intensity ratio between stimulated and unstimulated cells set at 100% [24].

The functional effects of the *FAS* mutations were assessed by transfecting the mutated cDNA, subcloned into pcDNA3.1 (Invitrogen) in 293T cells. Wild-type cDNA of *FAS* bearing the FLAG tag at the 5′-end was a kind gift of Giovina Ruberti (National Research Council, CNR, Rome). The p.Gln273His and p.Glu261Lys mutants were created in the *FAS* construct by PCR. Cells were transfected and lysed as for Munc13-4 and immunoblotted with anti-FLAG mAb (Sigma).

### Analysis of caspase-8 activity

Caspase-8 activity was evaluated, as previously reported [25], in 100 µg of cell lysates obtained from Fas-transfected 293T cells (5 x 10^6^), 24 hours after transfection, using a fluorimetric assay according to the manufacturer’s instructions (MBL, Watertown, MA). The results were expressed as relative caspase-8 activity (in %), which was calculated as (activity of Fas transfected cells/activity of mock transfected cells) X100%.

### Functional analyses of patient NK cells

PBMC were cultured overnight at 37°C in 5% CO_2_ in media with or without rIL-2 (600 UI/ml) (Proleukin, Chiron Corp., Emeryville, USA) to test degranulation of resting and activated NK cells, respectively. PBMC derived from patients’ relatives and/or unrelated healthy donors were tested in parallel. Surface expression of CD107a was assessed on CD3^-^CD56^+^ cells upon co-incubation of PBMC with K562 cells in the presence of Phycoerythrin-conjugated anti-CD107a mAb for 2 hours at 37°C, as previously described [26,27]. Thereafter, cells were stained with APC-conjugated anti-CD56 and PerCP-conjugated anti-CD3 mAb, and analyzed by flow cytometry (FACSCalibur, Becton Dickinson Biosciences, CA, USA). The results were considered by assessing the change in % CD107a (i.e., % CD107a^+^ cells in stimulated samples – % CD107a^+^ cells in unstimulated samples). All reagents were from BD Biosciences. NK cells were also purified using the RosetteSep method (StemCell Technologies, Vancouver, British Columbia, Canada), following manufacturer’s instructions, and cultured in appropriate conditions to obtain high numbers of polyclonal activated NK cells [26]. To analyze the cytolytic activity in 4 hour ^51^Cr-release assays, PBMC were tested against K562 cells, while activated NK cells were tested against the HLA-class I-negative B-EBV cell line 721.221, as previously described; lytic units were calculated at 30% lysis [26,27].

### Statistical analysis

Statistical analysis was performed using the ANOVA followed by Dunnett’s multiple comparison test; *p<0.05, **p<0.01. The results are shown as the mean and standard error (SE). Genotype distributions were analyzed with the Fisher’s exact test. All *P*-values are 2-tailed, and the significance cut-off was *p*<0.05.

## Results

### Genetic analyses

**Figure 1:**
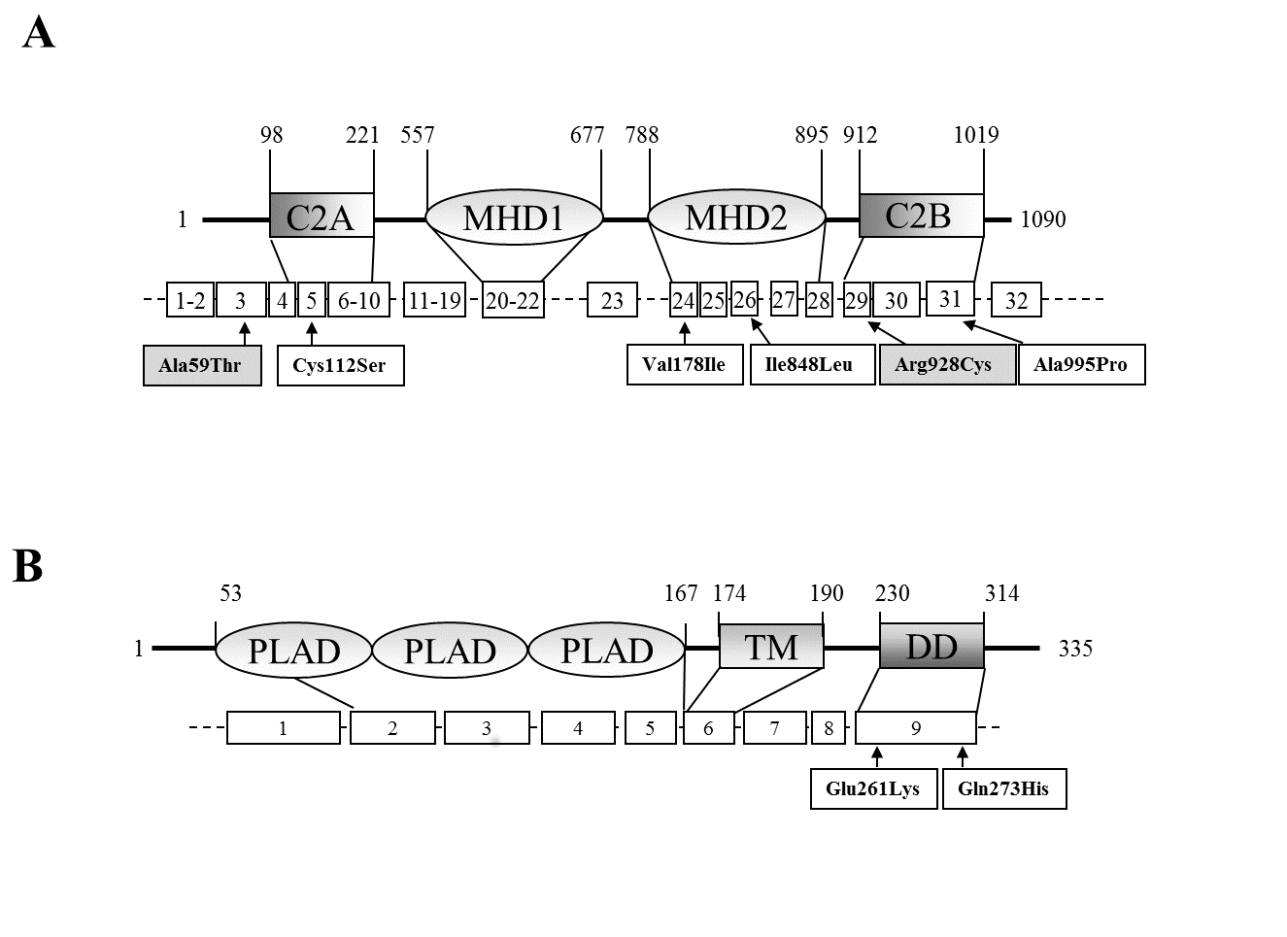
*UNC13D* and *FAS* variations carried by ALPS/DALD patients. Graphical representation (not in scale) of the Munc13-4 [A] and Fas [B] proteins (upper schemes: numbers indicate the amino acid positions) and genes (lower scheme: boxes represent the exons; arrows indicate the mutations). C2: C2 domain; MHD: Munc13-homology domain. PLAD: preligand assembly domain; TM: transmembrane domain; DD: death domain.

The coding sequences (exons and intron boundaries) of *UNC13D* were sequenced in 21 patients with ALPS (ALPS-FAS: N=7; ALPS-U:N = 14) and 20 with DALD. We identified 6 heterozygous missense variations in *UNC13D* in 8 patients (2 ALPS-FAS, 3 ALPS-U, 3 DALD). The variations and their inheritance are described in Table 1 and Figure 1.

**Table 1:** Gene *v*ariations detected in patients with ALPS or DALD.

**Patients (gender)**	**Diagnosis**	Fas function*			***FAS***		***UNC13D***	
	**Pt**	**Pt**	**F**	**M**	**Variation**	**Inh†**	**Variation**	**Inh†**
**Pt.1** (female)	ALPS-FAS	D	D	N	p.Gln273His	F	p.Arg928Cys	M
					(c.819G>C)		(c.2782C>T)	or F‡
**Pt.2** (male)	ALPS-FAS	D	ND	ND	p.Glu261Lys	F	p.Cys112Ser	M
					(c.755G>A)		(c.335C>G)	
**Pt.3** (female)	ALPS-U	D	D	D			p.Ala59Thr	M
							(c.175G>A)	
**Pt.4** (male)	ALPS-U	D	D	D			p.Ile848Leu	M
							(c.2542A>C)	
							p.Ala995Pro	M
							(c.2983G>C)	
**Pt.5** (female)	ALPS-U	D	ND	ND			p.Val781Ile	ND
							(c.2342G>A)	
**Pt.6** (male)	DALD	D	D	D			p.Ala59Thr	F
							(c.175G>A)	
**Pt.7** (female)	DALD	D	D	D			p.Arg928Cys	M
							(c.2782C>T)	
**Pt.8** (male)	DALD	D	D	ND			p.Arg928Cys	ND
							(c.2782C>T)	

* D = defectve, N = normal, Pt = patient, F = father, M = mother † Inheritance, F = father, M = mother; ND = not determined; no parent displayed ALPS, DALD, XLP, or FHL; Pt.1’s mother had rheumatoid arthritis. ‡ both parents carried the variation.

Two variations had been previously described in patients with FHL; two patients carried p.Ala59Thr (c.175G>A; rs9904366) and three p.Arg928Cys (c.2782C>T; rs35037984). Four other variations were identified, i.e., p.Cys112Ser (c.335G>C; rs141540493), p.Val781Ile (c.2342G>A; rs149871493), p.Ile848Leu (c.2542A>C; rs144968313), and p.Ala995Pro (c.2983G>C; rs138760432). These variations have been recently described in the dbSNP database as rare variants with an allele frequency of <0.01%, and each variant was found in a single patient. Pt. 4 carried two variations, p.Ile848Leu and p.Ala995Pro, inherited from the same parent.

To assess the variation frequency in the general populations and in subjects with a different autoimmune disease, we sequenced *UNC13D* in 61 healthy controls and 38 patients with MS. The results showed that p.Ala59Thr was found in 5 healthy controls and 3 MS patients, and p.Arg928Cys in 8 healthy controls and 2 MS patients. Moreover, one healthy control carried the novel variation p.Pro271Ser (c.811C>T), absent in the other groups (Table 2). Because p.Ala59Thr and p. Arg928Cys were detected in all patients and control groups with similar allelic frequencies, they were not further considered. Because p.Cys112Ser, p.Val781Ile, p.Ile848Leu, and p.Ala995Pro were detected in the ALPS group alone, we further assessed their frequency in the Italian population by genotyping them in 100 additional healthy controls. None of these variations were identified in the healthy controls, indicating that their allele frequency is relatively low.

**Table 2:** Missense variations detected in 21 ALPS, 20 DALD, 38 MS patients, and 61 healthy controls.

	**Functional effect^‡^**	**ALPS**	**DALD**	**Controls**	**MS**
		(N=42)*	(N=40)*	(N=122)*	(N=76)*
**Frequent variations^†^**					
Arg928Cys	Not performed	1	2	8	2
Ala59Thr	Not performed	1	1	5	3
*Total alleles with frequent variations*		2	3	13	5
					
**Private variations^†^**					
Ile848Leu^‡^	Loss-of-function^‡^	1	0	0	0
Ala995Pro^‡^	Loss-of-function^‡^	1	0	0	0
Cys112Ser	Loss-of-function	1	0	0	0
Val781Ile	Loss-of-function	1	0	0	0
Pro271Ser	Normal Function	0	0	1	0
*Total alleles with loss of function*		3	0	0	0
*p=0.03* ^*§*^					

* allele numbers † amino acid substitution ‡ carried in the same allele § P value vs Controls (Fisher exact test)

Of the five patients whose inheritance pattern of *UNC13D* variations could be determined, four (80%) were maternal and one (20%) was paternal (Table 1). To evaluate whether this apparent bias was due to genetic imprinting favoring expression of the maternal allele, we performed RT-PCR on mRNA derived from Pt.2, Pt.7, and two other patients who were heterozygous for the common synonymous polymorphism c.3198A>G (p.Gln1066Gln). Complementary DNA were then cloned, and 30 independent clones were sequenced for each patient. The results showed that both alleles were expressed at approximately the same levels in each subject, which did not support maternal genetic imprinting in these patients (data not shown).

### Functional analyses

**Figure 2:**
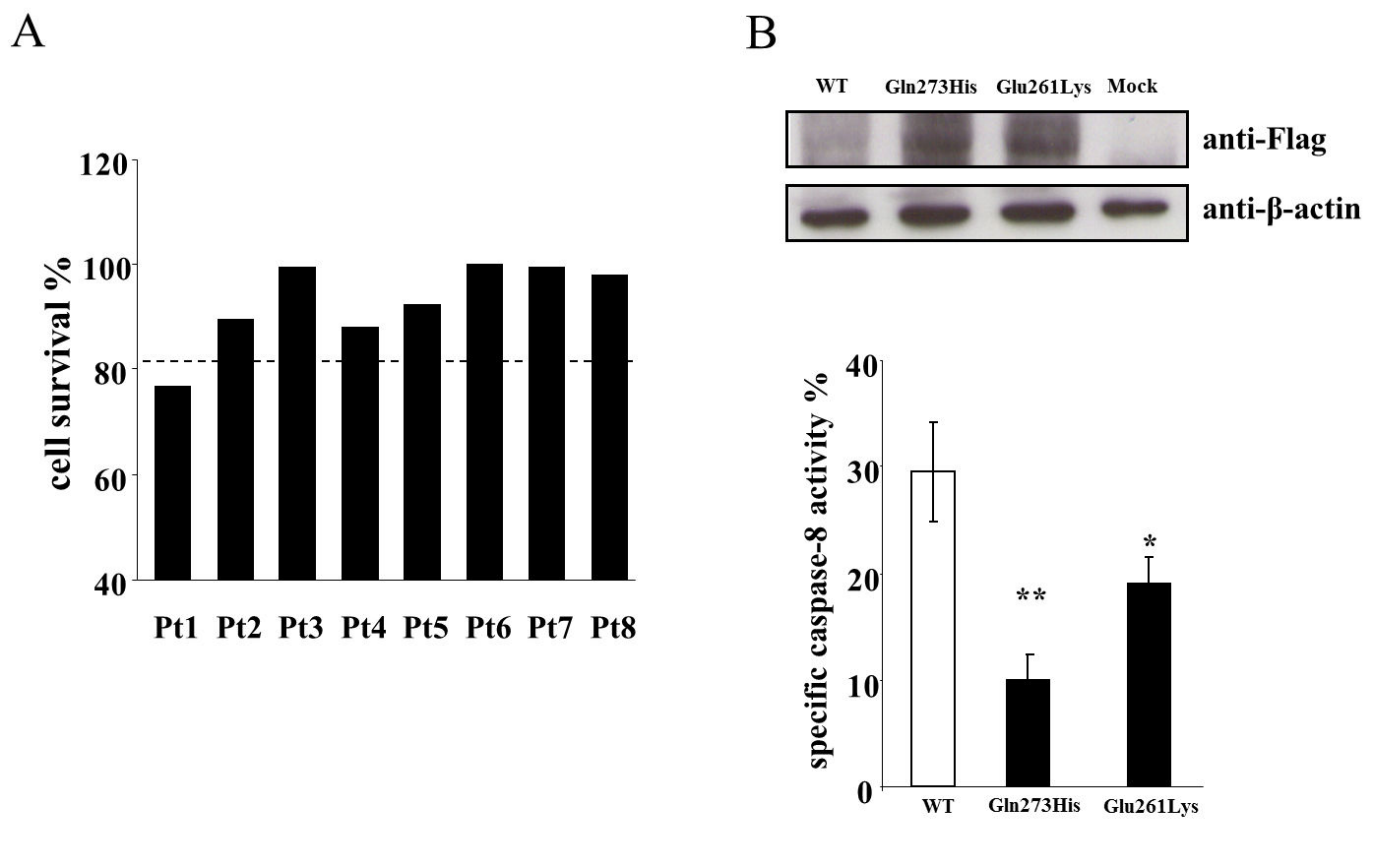
Defective Fas function in the ALPS and DALD patients carrying the UNC13D variations. [A] Fas-induced cell death in T cells from the ALPS and DALD patients carrying the *UNC13D* variations. Activated T cells were treated with anti-Fas mAb and survival was assessed after 18 hours. The results are expressed as specific cell survival %. The dotted line indicates the upper limit of the normal range calculated as the 95^th^ percentile of data obtained from 200 healthy controls; two or more were run in each experiment as positive controls; each patient was evaluated at least twice with the same result. [B] Fas expression and caspase-8 activity in lysates of 293T cells transfected with the wild-type (WT) or mutated form of *FAS* (Pt.1: p.Gln273His, Pt.2: p.Glu261Lys); cells were lysed 24 hours after transfection. *Upper panels*: Western blot analysis of the transfected Fas performed using anti-FLAG and anti-β-actin antibodies*. Lower panels*: fluorimetric enzyme assay for caspase-8 activity. Data are relative to those displayed by mock-transfected cells and are expressed as the mean and SE of the results from 4 experiments performed in duplicate. *p<0.05; **p<0.01 vs. Fas^wt^ transfected cells.

Fas-induced cell death assessed in T cells from the ALPS and DALD patients carrying the *UNC13D* variations is shown in Figure 2A. All patients displayed defective Fas function except for Pt.1, whose Fas function was considered as borderline with regard to statistical significance.

The *FAS* mutations present in Pt.1 and Pt.2 were p.Gln273His and p.Glu261Lys missense mutations, respectively. To assess their effect on Fas function, the wild-type (Fas^WT^) and mutated (Fas^Gln273His^, Fas^Glu261Lys^) forms of Fas cDNA were transfected into 293T cells. Twenty-four hours after transfection, 293T cells were harvested and caspase-8 activity was assessed in cell lysates. The results showed that caspase-8 activation was significantly lower in the cells transfected with Fas^Gln273His^ and Fas^Glu261Lys^ than in those transfected with Fas^WT^ (Figure 2B).

The effects of the *UNC13D* missense variations on Munc13-4 protein expression and NK function were evaluated in the PBMC of all patients. NK function was evaluated by assessing the cytotoxic activity of resting NK cells against K562 cells and that of activated NK cells against the HLA-class I-negative B-EBV cell line 721.221. Resting and activated NK cells were also tested for granule exocytosis, the most appropriate assay to detect Munc13-4 defects [28]. The results showed that values were in the normal range for all patients (data not shown), which was consistent with previous data on donors carrying heterozygous mutations of *UNC13D*.

**Figure 3:**
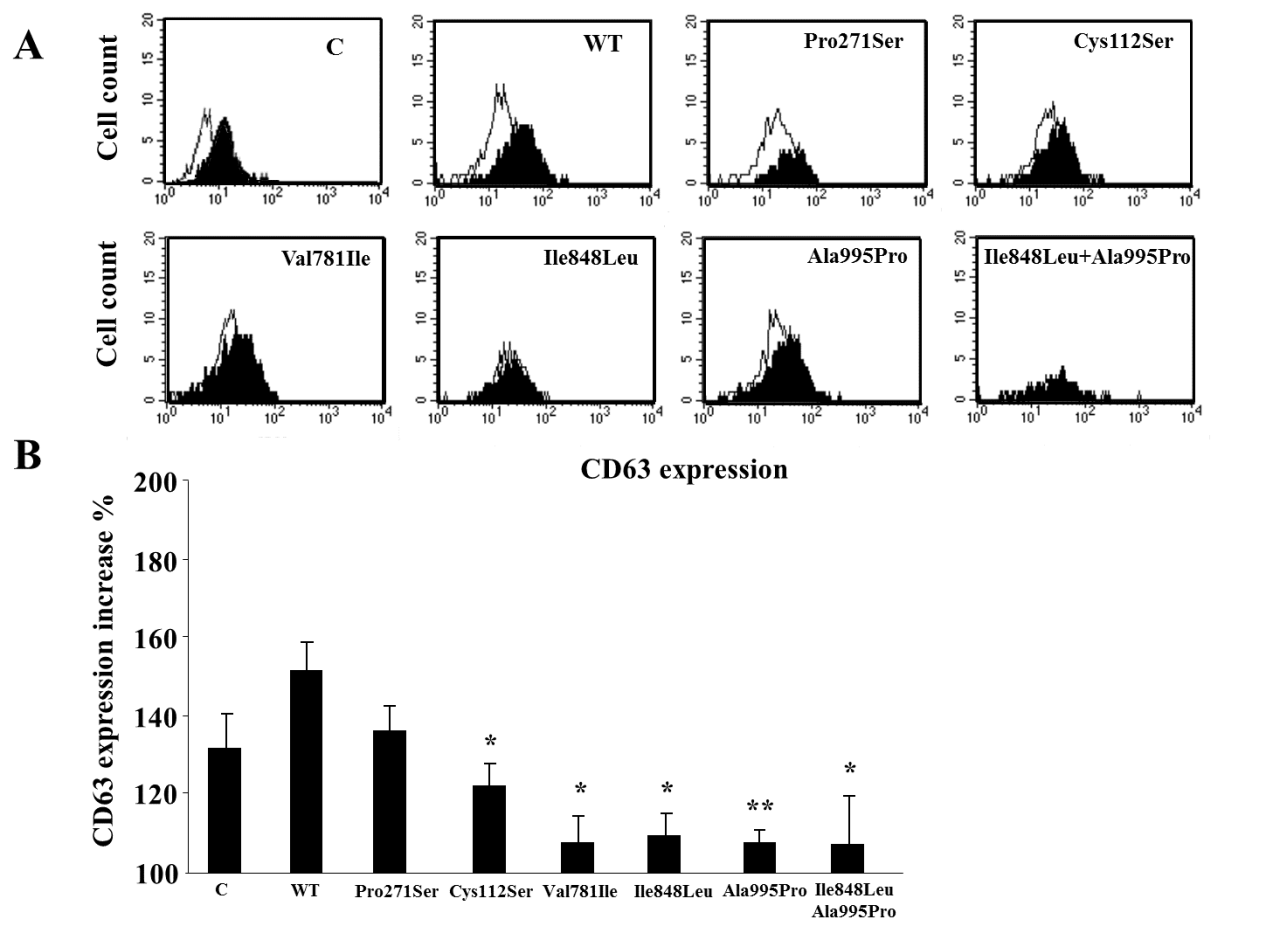
Functional effect of the “private” missense variations of *UNC13D*. HMC-1 cells were transiently transfected with wild-type (WT) *UNC13D* and mutated forms carrying the (p.Cys112Ser, p.Val781Ile, p.Ile848Leu, p.Ala995Pro, p. Ile848Leu/p.Ala995Pro, and p.Pro271Ser) variations (C = untransfected cells). Twenty-four hours after transfection, cells were teated (or not) for 10 min with fMLP, and expression of CD63 was evaluated by flow cytometry. [A] Cytofluorimetric histograms of CD63 expression in fMLP-stimulated (black) and unstimulated (white) cells transfected with each construct; one experimental representative of six is shown. [B] Mean and SE of the fMLP-induced expression of CD63 from six experiments; results are relative to the CD63 expression displayed by unstimulated cells (set at 100%) in each experiment; the asterisk marks the statistically significant difference versus cells transfected with the WT form; *p<0.05; **p<0.01 vs MUNC^wt^ transfected cells.

To further assess the functional effect of the *UNC13D* variations detected in one subject only, they were inserted into a cDNA encoding *UNC13D* fused to the SV5 epitope tag. The Munc13-4^wt^, Munc13-4^Cys112Ser^, Munc13-4^Val781Ile^, Munc13-4^Ile848Leu^, Munc13-4^Ala995Pro^, Munc13-4^Ile848Leu/Ala995Pro^, and Munc13-4^Pro271Ser^ constructs were transfected into the HMC-1 mastocytoma cell line. Western blot analysis showed that all constructs were expressed at similar levels indicating that the polymorphisms did not have a substantial effect on Munc13-4 expression (data not shown). To assess the effect of these variations on Munc13-4 function, we evaluated the capacity of fMLP to induce secretory granule fusion with the plasma membrane in HMC-1 cells. Fusion was monitored by an increase in CD63 expression on the cell surface. Figure 3 shows that fMLP increased surface expression of CD63 by similar amounts in the cells transfected with Munc13-4^wt^ or Munc13-4^Pro271Ser^ constructs. In contrast, fMLP increased CD63 expression to a significantly lower extent in cells that have been transfected with the Munc13-4^Cys112Ser^, Munc13-4^Val781Ile^, Munc13-4^Ile848Leu^, Munc13-4^Ala995Pro^, and Munc13-4^Ile848Leu/Ala995Pro^ constructs (*p<0.05).

## Discussion

Munc13-4 is a member of the Munc13-like family of proteins. It is highly expressed in CTL, NK cells, and mast cells and it is involved in granule exocytosis. Once granules are tethered to the plasma membrane, a priming step is required to enable fusion of the granule membrane with the plasma membrane. In this priming step, granules interact with a docking complex composed of Munc18-2 and Syntaxin-11. Thus, Munc13-4 triggers the switch of syntaxin-11 from a closed to an open conformation enabling fusion [29].

The present study detected six missense variations of *UNC13D* in ALPS-FAS (2/7, 29%), ALPS-U (3/14, 21%), and DALD (3/20, 15%) patients. Among them, two (p.Ala59Thr, p.Arg928Cys) had been previously reported in FHL3, whereas the other four (p.Cys112Ser, p.Val781Ile, p.Ile848Leu, p.Ala995Pro) were reported in the dbSNP database as rare variations with unknown functional and pathological significance. Moreover, both Ile848Leu and Ala995Pro have been described in *cis* in one patient with systemic Juvenile Idiopathic Arthritis (SJIA) and patients with FHL [30].

Only p.Ala59Thr and p.Arg928Cys were found in more than one patient, with the former carried by an ALPS-U and a DALD patient, and the latter by an ALPS-FAS and two DALD patients. These p.Ala59Thr and p.Arg928Cys variations were detected in several healthy controls and MS patients with similar allelic frequencies (p.Ala59Thr: ALPS 2.4%, DALD 2.5%, healthy controls 4.1%, MS 4%; p.Arg928Cys: ALPS 2.4%, DALD 5%, healthy controls 6.5%, MS 2.6%). These data argue against substantial role for these variations in ALPS and DALD. The p.Ala59Thr variation had been previously reported in two families with FHL, but always in *cis* with a pathogenic mutation, making it difficult to assess its contribution to pathogenesis [31]. The p.Arg928Cys variation had been previously reported in FHL patients and a recent genotype-phenotype study detected it in 8 patients carrying biallelic *UNC13D* mutations from 7 unrelated families [27]; yet, some of these FHL3 patients had a third missense mutation too. However, it could be a mild variant whose effect could not be detected in the small groups of subjects used in our study.

The other four variations were carried by three ALPS patients and were absent in DALD and MS patients, and in the healthy controls. The p.Cys112Ser variation was detected in an ALPS-FAS patient who also carried a *FAS* mutation (p.Gln261Lys); the *FAS* and the *UNC13D* mutations were inherited from the father and the mother, respectively. The p.Ile848Leu and p.Ala995Pro variations were carried by an ALPS-U patient and were in *cis*, as previously reported in SIJA and FHL [30], because both of them were inherited from the mother. The p.Val781Ile variation was detected in an ALPS-U patient. These variations were located within key functional domains of Munc13-4, characterized by two C2 domains (C2A, C2B) separated by long sequences containing two Munc13-homology domains (MHD) [29,32–34]. C2 domains bind calcium ions and are involved in targeting proteins to cell membranes; MHD domains are essential for the cellular localization of Munc13-4. The p.Cys112Ser variation was located in the C2A domain, p.Val781Ile and p.Ile848Leu in the MHD2 domain, and p.Ala995Pro in the C2B domain. The effect of these variations was assessed upon transfection in the HMC-1 mast cell line, commonly used to study granule exocytosis, which showed that all of them significantly decreased Munc13-4 function as detected by decreased fMLP-induced granule exocytosis. By contrast, one further variation (p.Pro271Ser) detected in a healthy control was located in the C2A domain but functional analysis showed that it did not significantly decrease Munc13-4 function.

The loss of function effects detected in the transfected cells are in contrast with the normal NK activity detected in the PBMC of the patients carrying the p.Cys112Ser, p.Val781Ile, p.Ile848Leu, and p.Ala995Pro variation. This discrepancy may be ascribed to a difference in sensitivity of the two types of assays. The NK function assays can, in fact, detect severe defects displayed by subjects carrying biallelic loss of function mutations of *PRF1* or *UNC13D*, but not the mild defect displayed by their healthy parents carrying monoallelic mutations. By contrast, mild defects could be detected by our assay in HMC-1 cells in which transfection forces expression of high levels of the Munc13-4 variants.

These data showed that rare loss of function variations of *UNC13D* are observed in ALPS patients with a higher frequency (7%) than in the healthy control (0%), DALD (0%), and MS (0%) groups. Thus, these variations may have an impact in the development of ALPS. Support for this hypothesis comes from a patient who carried loss-of-function mutations in *FAS, UNC13D*, and *XIAP*. He was not included in this study because the genetic and clinical complexity of his picture fulfilled the diagnostic criteria of ALPS but also shared features of FHL and X-linked lymphoproliferative disease (Boggio E. et al, submitted).

Defective functions of Fas and Munc13-4 might cooperate in disrupting the ability of the immune system to shut off and interfere with the anti-viral response. These processes involve both Fas and NK/NKT cell function whose cytotoxicity is crucial for the clearance of virus-infected cells and the fratricide of activated immune cells [35]. Persistence of viral infection and an inability to switch off the immune response may contribute to the lymphocyte accumulation and the autoimmune reactions displayed by ALPS patients.

These data suggest that the *UNC13D* variations may be considered part of an oligogenic background, predisposing individuals to ALPS development. This may involve genes encoding for perforin (*PRF1*), osteopontin (*OPN*), and Signaling Lymphocyte Activation Molecule-Associated Protein (*SH2D1A*), whose variations have been suggested to be risk factors for ALPS or DALD development. From this perspective, patients 1, 3, 4, 5, 6, 7, and 8 also notably carried the c.1239A>C variation of *OPN*, associated with ALPS and DALD [36]. Further, patients 2 and 8 displayed hemizygosity for the -349T variation of *SH2D1A*, which has been associated with ALPS and DALD [37]. Finally, patient 7 also carried the p.Ala91Val variation of *PRF1*, which has been associated with DALD [22].

ALPS and DALD display a similar clinical picture and share an inherited defect of Fas function and the modifying effect exerted by variants of *OPN*, *PRF1*, and *SH2D1A*. However, DN T cell expansion is only present in ALPS, which may mark immunopathological differences because a direct role has been ascribed to these cells in ALPS development. This work shows that mutations of *UNC13D* may also represent an immunologic difference because they were detected in ALPS but not in DALD patients. Moreover, the *UNC13D* variations were not detected in MS patients, which suggested that they are not a common risk factor for autoimmunity. However, a possible role of *UNC13D* in development of autoimmune diseases other than ALPS has been previously suggested in patients with SJIA [30,38], who may also display decreased NK function [39]. By contrast, the *OPN* and *PRF1* variants were involved in development of several other autoimmune diseases [40–47]. Future whole-genome or exome sequencing studies will reveal the complex genetic scenario that may contribute to ALPS and DALD.
